# East Asian summer monsoon precipitation variability since the last deglaciation

**DOI:** 10.1038/srep11186

**Published:** 2015-06-18

**Authors:** Fahu Chen, Qinghai Xu, Jianhui Chen, H. John B. Birks, Jianbao Liu, Shengrui Zhang, Liya Jin, Chengbang An, Richard J. Telford, Xianyong Cao, Zongli Wang, Xiaojian Zhang, Kandasamy Selvaraj, Houyuan Lu, Yuecong Li, Zhuo Zheng, Haipeng Wang, Aifeng Zhou, Guanghui Dong, Jiawu Zhang, Xiaozhong Huang, Jan Bloemendal, Zhiguo Rao

**Affiliations:** 1 MOE Key Laboratory of Western China’s Environmental System, Lanzhou University, Lanzhou 730000, China; 2 Institute of Nihewan Archaeology Research, College of Resources and Environment, Hebei Normal University, Shijiazhuang 050024, China; 3 Department of Biology and Bjerknes Centre for Climate Research, University of Bergen, N-5020 Bergen, Norway; 4 Environmental Change Research Centre, University College London, London WC1E 6BT, UK; and School of Geography and the Environment, University of Oxford, Oxford OX1 3QY, UK; 5 State Key Laboratory of Marine Environmental Science, Xiamen University, Xiamen 361005, China; 6 Key Laboratory of Cenozoic Geology and Environment, Institute of Geology and Geophysics, Chinese Academy of Sciences, Beijing 100029, China; 7 Department of Earth Sciences, Sun Yat-Sen University, Guangzhou 510275, China; 8 Department of Geography and Planning, University of Liverpool, Liverpool L69 3BX, UK

## Abstract

The lack of a precisely-dated, unequivocal climate proxy from northern China, where precipitation variability is traditionally considered as an East Asian summer monsoon (EASM) indicator, impedes our understanding of the behaviour and dynamics of the EASM. Here we present a well-dated, pollen-based, ~20-yr-resolution quantitative precipitation reconstruction (derived using a transfer function) from an alpine lake in North China, which provides for the first time a direct record of EASM evolution since 14.7 ka (ka = thousands of years before present, where the “present” is defined as the year AD 1950). Our record reveals a gradually intensifying monsoon from 14.7–7.0 ka, a maximum monsoon (30% higher precipitation than present) from ~7.8–5.3 ka, and a rapid decline since ~3.3 ka. These insolation-driven EASM trends were punctuated by two millennial-scale weakening events which occurred synchronously to the cold Younger Dryas and at ~9.5–8.5 ka, and by two centennial-scale intervals of enhanced (weakened) monsoon during the Medieval Warm Period (Little Ice Age). Our precipitation reconstruction, consistent with temperature changes but quite different from the prevailing view of EASM evolution, points to strong internal feedback processes driving the EASM, and may aid our understanding of future monsoon behaviour under ongoing anthropogenic climate change.

Whether the Asian summer monsoon responds directly on an orbital time scale to Northern Hemisphere summer insolation forcing without phase lag^1^,^2^, or whether the response is significantly delayed by internal feedback forcings^3^,^4^,^5^, has been intensively debated. This is mainly due to the lack of reliable palaeoclimatic records with a combination of a robust chronology and an unequivocal monsoon proxy^6^,^7^,^8^. As a major component of the Asian summer monsoon system, EASM variability controls almost all aspects of hydrology and ecology of East Asia^9^,^10^. Through its anomalous precipitation behaviour causing severe floods or droughts, the EASM considerably influences the economic and societal activities of one-third of the world’s population^11^,^12^. In the last two decades, numerous studies have focused on the evolution of the EASM during the late Quaternary by investigating diverse natural archives such as aeolian sequences^13^,^14^, lake sediments^15^, peat deposits^16^, and cave deposits^2^,^17^,^18^. Among these studies, oxygen-isotope (δ^18^O) records from speleothems have been viewed as one of the most robust EASM records mainly because of their very precise age controls^17^,^18^. However, the interpretation of speleothem δ^18^O in China remains highly controversial. Recent observational analysis^6^,^7^ and isotope modelling studies^8^,^19^,^20^ suggested that speleothem δ^18^O in southern China does not reflect the local rainfall variability in the cave region of southern China. Rather, it is essentially a signal of precipitation isotopic composition, which is determined mainly by rainfall variability in the upstream region over the Indian Ocean and Indian Monsoon region^21^. Therefore, high-resolution reconstructions combining both a robust chronology and an unambiguous proxy which can help resolve the discrepancies between the different views of EASM evolution during the late Quaternary^2^,^22^ are urgently needed. In particular, the timing of the Holocene monsoon maximum, fundamental to revealing the phase relationship between the summer monsoon and insolation, is very contentious within the various reconstructions^15^,^17^,^23^,^24^. This impedes our full understanding of EASM behaviour and dynamics and limits our ability to predict EASM variability in the future, especially under a global warming scenario.

The EASM intensity can be directly represented by precipitation in North China: stronger (weaker) EASM circulation carries more (less) water vapour from the tropical Pacific and Indian Oceans, resulting in higher (lower) precipitation over North China^25^,^26^. Here we use a high-resolution, pollen-based quantitative precipitation reconstruction from a well-dated sediment core retrieved from an alpine lake in North China to assess EASM variability and to characterize the underlying dynamical mechanisms since the last deglaciation, during which major drivers of Earth’s climate system (e.g. insolation^27^, Atlantic Meridional Overturning Circulation (AMOC)^28^,^29^, El Niño/Southern Oscillation (ENSO)^30^) experienced dramatic changes from glacial to inter-glacial conditions.

Gonghai Lake (38°54' N, 112°14' E; 1,860 m above mean sea level), a hydrologically-closed alpine lake, is located on the NE margin of the Chinese Loess Plateau (Fig. 1), a typical EASM dominated region^13^,^14^,^23^,^24^. This montane area is well-suited for documenting natural climatic variability given the relatively limited human impact^31^, in contrast to the lowland plains and river valleys which have a long history of human activity. The current climate is dominated by the EASM with on average 77% of the annual 445 mm of precipitation occurring from June to September (Supplementary Fig. S1). Instrumental data indicate that precipitation at the site is representative of much of North China (Supplementary Fig. S2), and thus the site is ideal for assessing EASM variability. The regional vegetation is a transitional forest-steppe whose ecological variability is highly sensitive to precipitation changes, and past vegetation changes can reliably be reconstructed using pollen analysis. Our investigation of pollen assemblages and modern vegetation distribution suggests that the fossil pollen in the Gonghai Lake sediments mainly represents the vegetation composition of the area surrounding the lake, with only a minor contribution (<10%) from vegetation farther than 5 km from the lake (Supplementary Text S1). Given both the dominance of regional zonal vegetation (very few intra-zonal taxa) and the significant altitudinal zonation around the lake, the stratigraphic pollen data can be used to reflect regional vegetation succession and climate changes. Furthermore, results from a statistical approach for evaluating biotic-assemblage-based quantitative reconstructions^32^ demonstrate that the pollen record of Gonghai Lake is highly suitable for precipitation reconstruction (Supplementary Fig. S8).

We obtained a sediment core (GH09B) from the central part of Gonghai Lake and focus here on the upper 9.42-m which consist of fine-grained lacustrine sediments (see Methods and Supplementary Fig. S3). The age model is based on a combination of ^210^Pb/^137^Cs dating of the uppermost 0.35 m (Supplementary Fig. S4) and twenty-five accelerator mass spectrometry (AMS) ^14^C dates of terrestrial plant macrofossils (Supplementary Table S1 and Supplementary Fig. S5), all converted to calendar years using the calibration of Reimer *et al.*^33^ (see Methods and Supplementary Table S1). The Bayesian age-depth model indicates that sedimentation was continuous, with a mean sediment accumulation rate of 64.3 cm/ka spanning the last ca. 14.7 ka. We also validated the stratigraphy and chronology of core GH09B by comparing the lithology and dating results of parallel core GH09C from the same location (Supplementary Fig. S3; also see Methods).

Core GH09B was sub-sampled at a 1-cm interval (20-yr resolution on average) and analysed for pollen, magnetic susceptibility (MS) and calcium carbonate (CaCO_3_) content using standard procedures (see Methods). Pollen analysis was employed to document vegetation changes and quantitatively to reconstruct precipitation variability. The MS values of the Gonghai Lake sediments mainly reflect catchment surface stability generally controlled by regional environmental conditions^34^. CaCO_3_ content is considered as an indicator of changes in water column chemistry with respect to lake status, given the dearth of carbonate minerals in the Gonghai Lake catchment.

Pollen assemblages in core GH09B are dominated by temperate-forest (*Pinus, Picea, Betula, Quercus and Ulmus*) and temperate-steppe (*Artemisia*, Chenopodiaceae and Poaceae) pollen types (Supplementary Fig. S6 and Supplementary Text S2). The abundance of arboreal and herbaceous taxa exhibits large (~60–80%) and abrupt changes over the last 14.7 ka (Fig. 2a,b). The percentages of tree pollen were low and herb pollen percentages high during the last deglacial period, and vice versa during the early-mid Holocene. Broad-leaved tree pollen reached maximum abundance from ~7.8–5.3 ka (Fig. 2c). After 3.3 ka, tree pollen decreased continuously, whereas herb pollen increased. Using a calibration-function method^35^, the pollen data were transferred quantitatively into annual precipitation (P_ANN_). For this purpose, we used a modern surface pollen dataset consisting of 509 samples distributed along a large precipitation gradient (P_ANN_: 0–1000 mm; P_ANN_ at nearby Ningwu Station is 445 mm) and a small mean annual temperature gradient (T_ANN_: 3.6–8.6°C; T_ANN_ at Ningwu station is 6.1°C) to ensure reliable estimates of the precipitation optima and tolerances of the major pollen taxa, and hence a robust P_ANN_ reconstruction. In addition to statistical validation of the calibration function (the two component weighted-averaging partial least square (WAPLS) model featuring a coefficient of determination of 0.84 and a root mean square error of prediction (RMSEP) of 86 mm, see Methods and Supplementary Table S2), the close similarity of the reconstructed P_ANN_ to the instrumental P_ANN_ record for the most recent past (AD 1962–2008) (Supplementary Fig. S9) further demonstrates the robustness of our P_ANN_ reconstruction.

The time series of the pollen-based P_ANN_ portrays the evolution of the EASM since the last deglaciation (Fig. 2d). Compared to modern instrumental P_ANN_ (445 mm), a gradual 50% increase in precipitation from 380 mm at 14.7 ka to 570 mm at 7.0 ka indicates a continuously intensifying EASM across the late deglacial-Holocene transition. The maximum monsoon intensity, with an average P_ANN_ of 574 mm, ~30% higher than the modern value, occurred from 7.8 to 5.3 ka. A two-step gradual decrease in monsoon intensity commenced at 5.3 ka and 3.3 ka, with a more prominent decline at 3.3 ka (Supplementary Fig. S12) which continued until the end of the Little Ice Age (LIA). These long-term monsoon trends inferred from our P_ANN_ model are generally consistent with the MS and CaCO_3_ records (Fig. 2e,f), which suggest a stable lake and surrounding catchment with a high lake level during the early and mid-Holocene (~11.5–3.3 ka).

Our P_ANN_ reconstruction documents the major sub-orbital scale global climatic events since the last deglaciation [Fig. 3g (blue curve)]. EASM precipitation generally decreased during cold climatic episodes, and increased during warm events. For example, following the warm Bølling/Allerød interstadial, a ~100 mm decrease in precipitation from ca. 12.8–11.7 ka indicates a weakened EASM [Fig. 3g (blue curve)] during the Younger Dryas (YD) cold event within the limits of the available chronology, when the Northern Hemisphere temperature was significantly reduced^36^ [Fig. 3e (magenta curve)]. A further precipitation decrease from 9.5 to 8.5 ka in the early Holocene corresponds to the widespread cold episode inferred from marine proxies associated with weakened thermohaline circulation^37^. The weakest Holocene EASM occurred during the LIA, when the monsoon precipitation was about 100 mm less (~20%) than during the Medieval Warm Period (MWP) [Fig. 3g (blue curve)]. The increased precipitation during the last ~150 years [Fig. 3g (blue curve)], since the end of the LIA, which is evident in our reconstructed precipitation record and is supported by a recent tree-ring precipitation reconstruction^38^, is consistent with the Northern Hemisphere warming trends inferred from proxy-based surface temperatures^39^. The synchroneity of these EASM responses and global climatic events further confirms the sensitivity of Gonghai Lake records to EASM variability in North China.

Previous proxy records from northern China may be unable fully to constrain the timing and amplitude of EASM variability in a detailed and precise way, given that many of them suffer from problems of limited temporal resolution, possible dating errors or ambiguous proxies^4^,^7^,^8^,^24^. However, those records can be used to carry out general comparisons with our P_ANN_ reconstruction on a multi-millennial timescale. A stronger EASM from ~8 to 3 ka, as indicated by the highest and the lowest contents of tree and herb pollen, respectively (Fig. 2a,b), with around 550 mm of reconstructed P_ANN_ [Fig. 3g (blue curve)], is supported by the increased frequency of palaeosol development from ~8.6–3.2 ka in the Chinese Loess Plateau (Fig. 3d), and decreased aeolian-sand activity from ~8.6–3.2 ka in the four main sandlands located to the west and NE of Gonghai Lake^40^. In addition, a vegetation record from Daihai Lake (~200 km to the north of Gonghai Lake; Fig. 1) clearly demonstrates that the wettest climate and strongest summer monsoon occurred during the mid-Holocene^15^ (Fig. 3c). Further evidence for a mid-Holocene EASM maximum comes from Qinghai Lake, ~1000 km to the west of Gonghai Lake (Fig. 1), where pollen data suggest a mid-Holocene climatic optimum^41^ (Fig. 3b). A generally decreasing trend of precipitation since ~6 ka is also documented in Tianchi Lake on the Chinese Loess Plateau^42^ [Fig. 3g (green curve)]. Furthermore, the mid-Holocene maximum EASM inferred from our reconstruction from Gonghai Lake corresponds to significant Neolithic cultural developments in northern China from ~7–5 ka, the period when the Yangshao Culture attained maximum prosperity compared to other Neolithic cultures before and after (Supplementary Fig. S10 and Supplementary Text S3). This suggests that the high precipitation [~160 mm higher vs. modern value; Fig. 3g (blue curve)] associated with an intense EASM during the mid-Holocene may have provided a favourable environment for cultural development in semi-arid North China. These various lines of evidence support the contention that our quantitative precipitation reconstruction indeed represents precipitation change over a large area of northern China, and that it reliably documents the Holocene EASM evolution with maximum monsoon precipitation occurring in the mid-Holocene.

The foregoing highlights the complexity of monsoon dynamics since the last deglaciation, because the EASM was not driven simply by changing insolation, as inferred from speleothem isotope records^2^. Although the EASM intensity tracks the insolation curve, the deviation between them during the early-Holocene suggests a ~4 ka delayed response to the summer insolation peak at ~10 ka, which we attribute to the remnant melting Laurentide ice sheet which delivered continuous freshwater input to the North Atlantic until ~7 ka^26^,^43^,^44^,^45^. The linkage between North Atlantic cold events and a weak Asian summer monsoon on centennial-millennial time scales since the last glacial has been suggested by various proxy records^17^,^46^,^47^. As our simulation results demonstrate, an anomalous freshening of the North Atlantic and the resultant weakened AMOC could have strongly depressed the insolation-driven EASM (Fig. 4a) through the strengthening of westerly and northerly winds (Supplementary Fig. S11). This would have caused the EASM to be weaker during the early Holocene than during the mid-Holocene (Fig. 4b), but still to be stronger than at present (Fig. 4c). Such a mechanical connection is also supported by present-day climate simulations showing that a reduction in the AMOC causes northerly surface wind anomalies over East Asia^48^. It is noteworthy that two intervals of significantly weakened millennial-scale EASM precipitation [Fig. 3g (blue curve)], during the YD and from 9.5–8.5 ka, correspond well to a weakened AMOC^28^,^29^.

After around 7 ka, the EASM strength changed broadly in response to orbitally-induced insolation forcing^27^ [Fig. 3g (orange curve)] because the glacial boundary conditions can be regarded as similar to the modern state, given both the dramatically decelerated rates of eustatic sea-level rise and the generally stable global ice volume after that time^49^,^50^. However, different from the gradually decreasing insolation during the late Holocene, the EASM experienced an abrupt decline at 3.3 ka [Fig. 3g (blue curve) and Supplementary Fig. S12], which may be partly attributed to the influence of tropical ocean conditions. During El Niño events, the tropical eastern Pacific Ocean warming increases tropospheric temperature and induces an eastward propagating Kelvin wave, leading to an increase in the tropospheric temperature in the western Pacific Ocean^51^,^52^, which weakens the land–sea thermal contrast and thus the EASM^53^. Both a paleo-ENSO reconstruction^30^ and our simulation indicate that the late Holocene exhibited an increased frequency of El Niño events (Supplementary Fig. S13), favouring a weak EASM (*r* = −0.50).

Our demonstration of a ~4 ka delay in the response of the maximum monsoon intensity to the Northern Hemisphere summer insolation maximum indicates that the prevailing view of an early Holocene EASM maximum inferred from speleothem oxygen isotope records in southern China^17^,^54^ (Fig. 3a) should be significantly revised. Our comparative study of the EASM behaviour in the context of coeval North Atlantic and tropical ocean climate records indicates a complex relationship between the EASM, the external forcing factor (Northern Hemisphere summer insolation), and internal feedback mechanisms which involve both high-latitude (AMOC) and low-latitude (ENSO) processes. We anticipate that our findings may improve the predictive ability of future model-based precipitation simulations. In addition, the overall positive relationship demonstrated here between temperature^36^,^55^ (Fig. 3e) and EASM precipitation [Fig. 3g (blue curve)], both on orbital and suborbital timescales, has significant implications for EASM behaviour under ongoing global warming, although the different responses of EASM to natural and anthropogenic forcing^56^ warrant further study.

## Methods

### Study area

**Figure 1:**
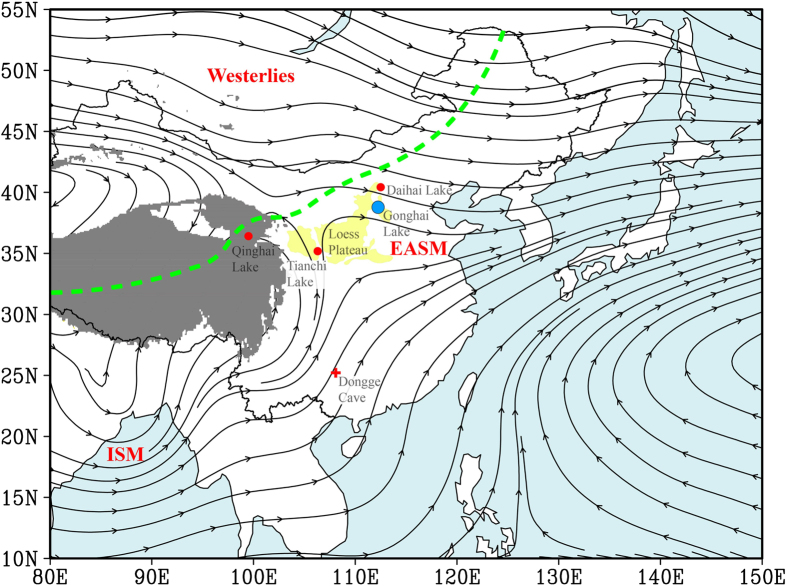
Summer (June-July-August, JJA) mean 700 hPa streamline based on NCEP/NCAR Reanalysis during 1971–2000. Blue dot indicates the location of Gonghai Lake. ‘EASM’, ‘ISM’, and ‘Westerlies’ denote the regions mainly influenced by the East Asian Summer Monsoon, the Indian Summer Monsoon, and the Westerlies, respectively. The modern Asian summer monsoon limit is shown by a green dashed line. Areas above 3000 m (above sea level) are shaded in grey. The Chinese Loess Plateau is shaded in light yellow. Daihai, Qinghai and Tianchi Lakes (red dots) and Dongge Cave (red cross) are additional key sites mentioned in the text. The figure was generated using GrADS v1.5.1.12^69^.

Gonghai Lake (38°54′N, 112°14′E; 1860 m above mean sea level) is located in Ningwu County, Shanxi Province, on the northern margin of the Chinese Loess Plateau (Fig. 1). It is a freshwater alpine lake formed on a plateau of the watershed between the Sanggan and Fenhe rivers. The basin is hydrologically-closed and the water source is mainly from precipitation. The surface area is ~0.36 km^2^ and the maximum water depth is ~10 m; the lake floor has a flat topography. The area is located in a transitional zone between semi-arid and semi-humid conditions, on the fringe of the modern Asian summer monsoon. The mean annual precipitation in Ningwu is 445 mm, of which, 77% occurs from June to September. The regional vegetation in the surrounding Lvliang Mountains is dominated by *Larix principis-rupprechtii*, *Pinus tabulaeformis* and *Populus davidiana* forest, while on the plateau, *Hippophae rhamnoides* scrub, *Bothriochloa ischaemum* grassland and *Carex* spp. are widely distributed. Documentary evidence suggests that intensive deforestation in this region did not occur until ~600 cal yr BP (Ming Dynasty)^31^. The exposed bedrock of the zonal ground surfaces was mainly formed during the Archaean to Cenozoic, and is comprised mainly of sandstone.

### Sediment lithology

Core GH09B extended to impenetrable substrate at a depth of 9.80 m. The interval 9.80–9.42 m consists of sand and gravel. The upper 9.42 m consists of lake sediment which can be divided into three lithological units: Unit 1 (9.42–7.70 m) consists of alternating layers of dark, silty clay and light–dark silt with the occasional occurrence of yellow sand. Unit 2 (7.70–2.60 m) consists of dark organic mud with light grey mud between 4.70 m and 2.60 m. Unit 3 (2.60–0.00 m) is dominated by silty clay with occasional plant macrofossils.

### Analytical methods

The bulk magnetic susceptibility (MS) of cores GH09B and GH09C was measured at a 1-cm interval using a Bartington MS2 magnetic susceptibility meter and MS2B sensor (Supplementary Figs. S3D and S3E). 771 samples, at ~1-cm interval, were analysed for pollen. ~1 g of sediment was treated using a modified HCl-NaOH-HF procedure^57^. One tablet of *Lycopodium* spores was added to each sample in order to estimate the pollen concentration. More than 500 terrestrial pollen grains were counted for each sample, and 81 pollen taxa were identified, including aquatic pollen grains, and spores. The sum of total terrestrial pollen was used as the denominator when calculating pollen percentages. Identifications are based on the Pollen Flora of China^58^ assisted by modern reference collections from Hebei Normal University. Pollen diagrams were plotted using Tilia v2.0.b.4^59^ and pollen-assemblage zones were constructed using stratigraphically-constrained cluster analysis (CONISS)^60^.

### Chronology

The chronology of the top 35 cm of core GH09B was obtained using a combination of ^210^Pb and ^137^Cs dating (Supplementary Fig. S4). Eighteen terrestrial plant macrofossil samples from core GH09B and seven terrestrial plant-macrofossil samples from core GH09C were measured for radiocarbon (^14^C) at the Xi’an AMS Laboratory, China, and at Beta Analytic Inc., Florida, USA. The MS records of these two cores correlate well and therefore the ^14^C sample depths of core GH09C were transferred to the corresponding depths of core GH09B, using magnetic susceptibility correlations. Thus we developed for the first time an exceptionally high-resolution age model for northern Chinese lakes. All conventional ^14^C ages were calibrated to calendar years using the IntCal09 calibration curve^33^. Supplementary Table S1 lists all measured and calibrated ages. The ages are expressed in years before present (BP) where “present” is defined as AD 1950. Bayesian age-depth modelling for Core GH09B (Supplementary Fig. S5) was performed using OxCal v4.2.2 and a Poisson-process (P-sequence) single depositional model at 1-cm increment with a K value of 100^61^.

### Quantitative reconstruction of precipitation

**Figure 3:**
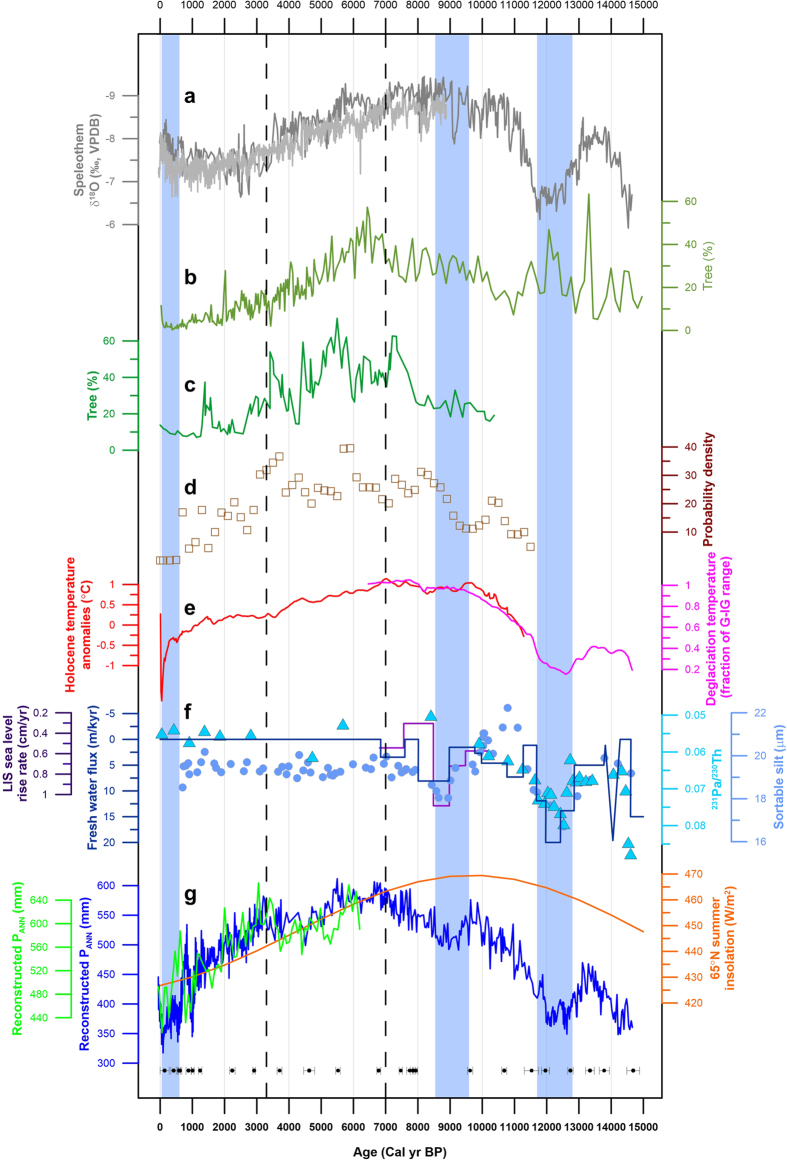
Comparison of Gonghai Lake EASM record with various other regional and global environmental signals. (**a**) Dongge cave speleothem δ^18^O records^17^,^54^. (**b**) Tree pollen percentages from Qinghai Lake^41^. (**c**) Tree pollen percentages from Daihai Lake^15^. (**d**) Frequency distribution of Chinese Loess Plateau palaeosol dates^40^. (**e**) Synthesized Northern Hemisphere (30°–90°N) temperature record during the last deglaciation^36^ (magenta line) and Holocene^55^ (red line). (**f**) Western subtropical Atlantic ^231^Pa/^230^Th record^28^ (blue circles) and northeast Atlantic sortable silt record^29^ (cyan triangles), both of which may indicate AMOC strength, and synthesized meltwater flux in the Northern Hemisphere^26^ (blue line) and the rate of sea-level rise from the Laurentide Ice Sheet^44^ (purple line), demonstrating continuous freshwater input during the last deglaciation and early Holocene, with the intervals of rapid melting during the YD and from 9.5 to 8.5 ka. (**g**) Pollen-based annual precipitation (P_ANN_) reconstructed from Gonghai Lake (blue line, this study) and similar reconstruction for the past 6 ka from the nearby Tianchi Lake^42^ (green curve) together with 65°N summer insolation^27^ (orange line). Black dots at the bottom are twenty-five AMS ^14^C dates from terrestrial-plant macrofossils with an uncertainty interval of 1σ. The shaded blue bars indicate periods of significantly decreased precipitation. The dashed lines indicate changes in the factors forcing EASM variability.

A modern calibration set of 1,860 surface pollen samples was used after excluding samples from tropical and arid regions, and those from sites potentially influenced by human activity, from the total of 2,689 samples^62^,^63^,^64^,^65^. The reconstruction was refined by limiting the mean annual temperature (T_ANN_) range to obtain a more robust pollen-based P_ANN_ calibration function, given that temperature and precipitation are the most significant variables controlling regional vegetation and that the target variable of this reconstruction is precipitation. A subset of 509 samples (Supplementary Fig. S7) within the T_ANN_ range 3.6–8.6 °C, which were ±2.5 °C from T_ANN_ of the nearest Ningwu Station, was used. The range of P_ANN_ is relatively large, from 0 to 1,000 mm. Long compositional gradient lengths (axis 1 = 3.4 standard deviation (SD) units of compositional turnover, axis 2 = 3.6 SD units) in the pollen data set suggested a unimodal-based method for regression and calibration^66^. A two component WAPLS model exhibiting the strongest statistical performance (*r*^2^ = 0.84, RMSEP = 86 mm) (Supplementary Table S2) was chosen for quantitative precipitation reconstruction^66^. In addition, a novel method was employed to assess the statistical significance of the reconstruction using both the modern and fossil data^32^. This showed that the P_ANN_ reconstruction based on the WAPLS-2 model and stratigraphical pollen data from core GH09B explains more of the variation in the fossil data than 99% of reconstructions (*p* = 0.001) derived from calibration-functions trained on random environmental data, whereas the T_ANN_ reconstruction is not statistically significant (*p* = 0.434) (Supplementary Fig. S8). In addition, this method was employed to reconstruct P_ANN_ changes using pollen percentage data from Tianchi Lake over the past 6 ka^42^, plotted in Fig. 3g (green curve). The best-performing two-component WAPLS model based on 525 modern samples has an *r*^2^ of 0.87 and a RMSEP of 74.7 mm [99% confidence level (*p* = 0.01)].

### EASM simulation experiments

The Kiel Climate Model (KCM), a coupled atmosphere–ocean–sea-ice general circulation model (AOGCM), was used to model the EASM from 9.5 ka to 0 ka with 10-times acceleration, as detailed in Lorenz and Lohmann^67^. Prior to the transient simulation (HT), two 1,000-yr control simulations for the pre-Industrial period (AD 1800, H0K) and the early Holocene (9.5 ka, H9K) were performed. The HT simulation was then started at the end of the H9K equilibrium run with transient orbital parameters. Supplementary Table S3 summarizes the boundary conditions adopted for these three simulations. A series of sensitivity simulations (hereafter PI, Exp_6 ka, Exp_8.5 ka, Exp_8.5 ka_ICE,_ and Exp_8.5 ka_MELTICE_) were performed following the description in Jin *et al.*^68^ with the Community Climate System Model 3 (CCSM3), which is also an AOGCM. PI, Exp_6 ka and Exp_8.5 ka are control runs for AD 1800, 6 ka and 8.5 ka, with corresponding orbital parameters and greenhouse gas concentrations. Additional forces were implanted in Exp_8.5 ka_ICE,_ and Exp_8.5 ka_MELTICE_. Supplementary Table S4 summarizes the boundary conditions adopted for these five simulations.

## Additional Information

**How to cite this article**: Chen, F. *et al.* East Asian summer monsoon precipitation variability since the last deglaciation. *Sci. Rep.* **5**, 11186; doi: 10.1038/srep11186 (2015).

## Supplementary Material

Supplementary Information

## Figures and Tables

**Figure 2:**
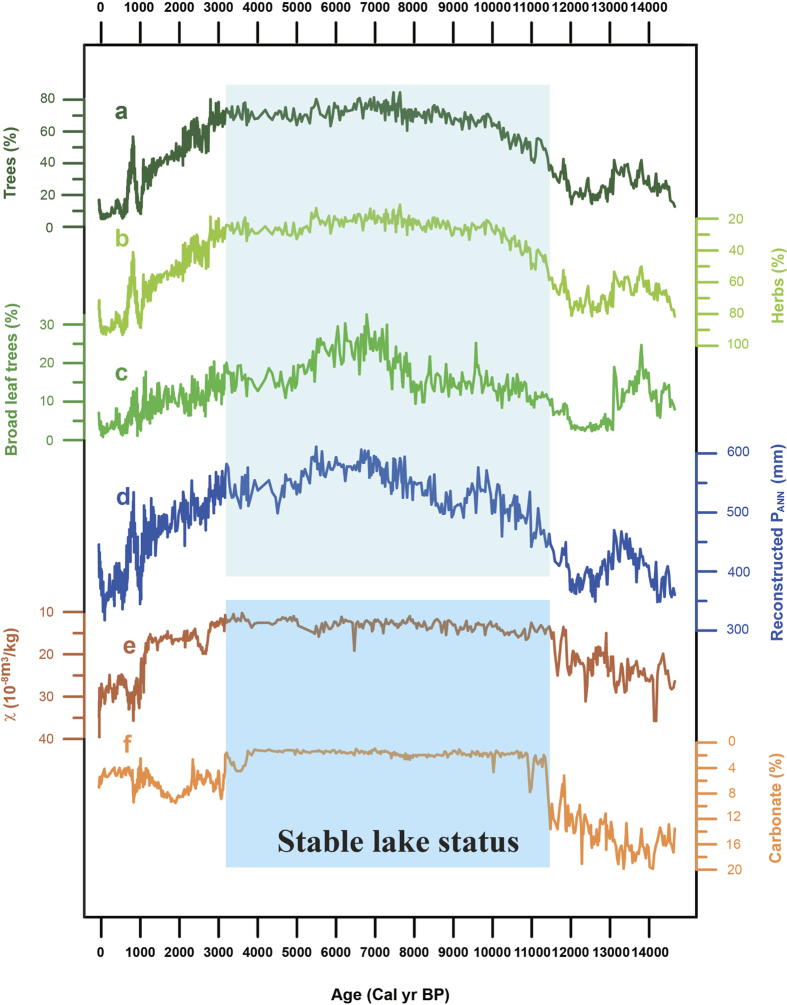
Variation in the reconstructed annual precipitation and other environmental proxies since 14.7 ka from Gonghai Lake, North China. (a) Percentage of tree pollen (%). (b) Percentage of herb pollen (%). (c) Percentage of broad leaf tree pollen (%). (d) Annual precipitation reconstructed by a calibration function applied to the fossil pollen assemblages. (e) Magnetic susceptibility. (f) Carbonate content. Shaded area from ~11.5–3.3 ka denotes an interval of stable lake status and maximum vegetable cover in the Gonghai Lake catchment.

**Figure 4:**
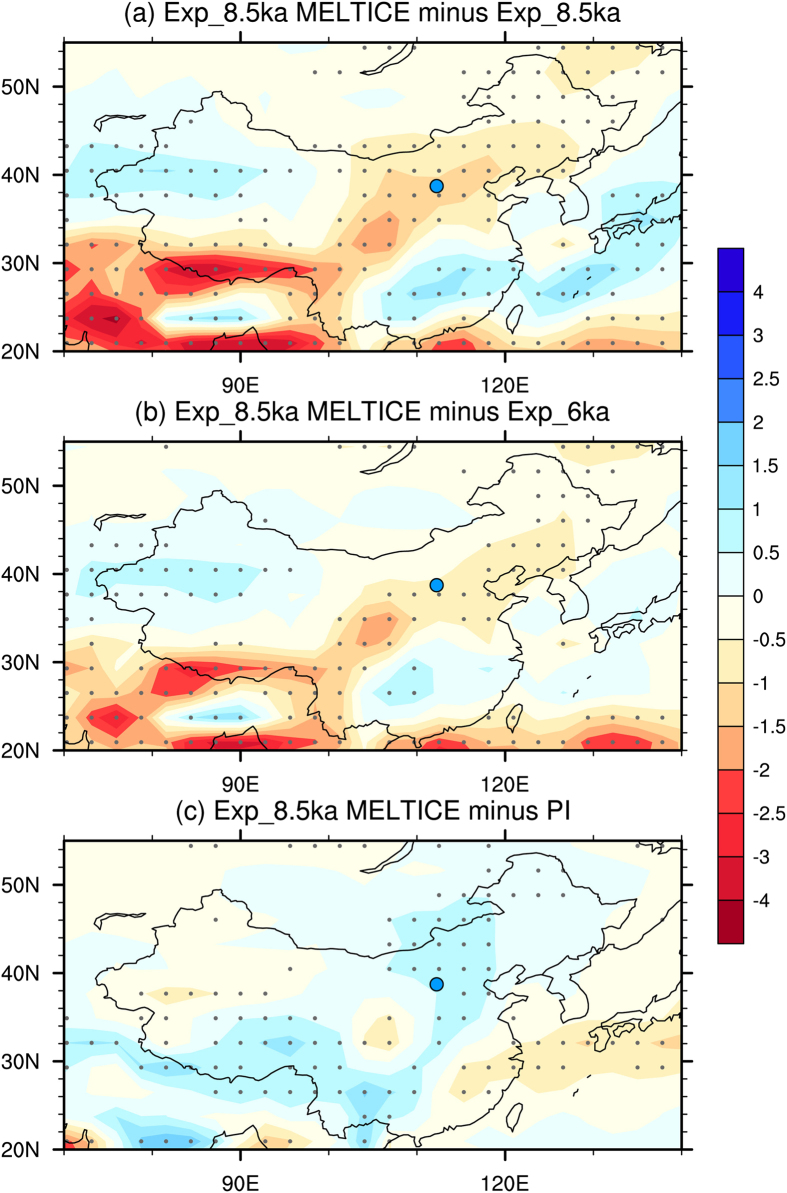
Summer precipitation changes (in mm/day) in sensitivity experiments (refer to Table S4 for detailed information). The results show the weakening of the EASM at 8.5 ka in northern China when considering glacial boundary conditions (**a**). The weakened EASM at 8.5 ka was weaker than at 6 ka (**b**) but stronger than the present (**c**). Blue dot indicates the location of Gonghai Lake. Dotted areas indicate changes which are significant at the 95% confidence level.

## References

[b1] Kutzbach J. E., Liu X., Liu Z. & Chen G. Simulation of the evolutionary response of global summer monsoons to orbital forcing over the past 280,000 years. Clim. Dyn. 30, 567–579 (2008).

[b2] Wang Y. J. *et al.* Millennial-and orbital-scale changes in the East Asian monsoon over the past 224,000 years. Nature 451, 1090–1093 (2008). 18305541 10.1038/nature06692

[b3] Overpeck J., Anderson D., Trumbore S. & Prell W. The southwest Indian Monsoon over the last 18000 years. Clim. Dyn. 12, 213–225 (1996).

[b4] Clemens S. C., Prell W. L. & Sun Y. Orbital–scale timing and mechanisms driving Late Pleistocene Indo–Asian summer monsoons: Reinterpreting cave speleothem δ^18^O. Paleoceanography 25, PA4207, doi:10.1029/2010PA001926 (2010).

[b5] An Z. S. *et al.* Glacial-interglacial Indian summer monsoon dynamics. Science 333, 719–23 (2011). 21817044 10.1126/science.1203752

[b6] Maher B. A. & Thompson R. Oxygen isotopes from Chinese caves: records not of monsoon rainfall but of circulation regime. J. Quat. Sci. 27, 615–624 (2012).

[b7] Tan M. Circulation effect: response of precipitation δ^18^O to the ENSO cycle in monsoon regions of China. Clim. Dyn. 42, 1067–1077 (2014).

[b8] Caley T., Roche D. M. & Renssen H. Orbital Asian summer monsoon dynamics revealed using an isotope-enabled global climate model. Nat. Commun. 5, doi: 10.1038/ncomms6371 (2014). 10.1038/ncomms6371 25373794

[b9] Wang B. The Asian Monsoon (Springer, Berlin, 2006).

[b10] Clift P. D. & Plumb R. A. The Asian monsoon: causes, history and effects (Cambridge University Press, 2008).

[b11] Huang R. H., Xu Y. H., Wang P. F. & Zhou L. T. The features of the catastrophic flood over the Changjiang River Basin during the summer of 1998 and cause exploration. Clim. Environ. Res. 3, 300 313 (1998) (In Chinese).

[b12] Fu C. B. & Ma Z. G. Global change and regional aridification. Chinese J. Atmos. Sci. 32, 752–760 (2008) (In Chinese).

[b13] An Z. S. *et al.* The long-term paleomonsoon variation recorded by the loess–paleosol sequence in central China. Quat. Int. 7-8, 91–95 (1990).

[b14] Zhou W. J. *et al.* Reappraisal of Chinese Loess Plateau stratigraphic sequences over the last 30,000 years-Precursors of an important Holocene monsoon climatic event. Radiocarbon 40, 905–913 (1998).

[b15] Xiao J. L. *et al.* Holocene vegetation variation in the Daihai Lake region of north-central China: a direct indication of the Asian monsoon climatic history. Quat. Sci. Rev. 23, 1669–1679 (2004).

[b16] Hong Y. T. *et al.* Inverse phase oscillations between the East Asian and Indian Ocean summer monsoons during the last 12000 years and paleo-El Niño. Earth Planet. Sci. Lett. 231, 337–346 (2005).

[b17] Wang Y. J. *et al.* The Holocene Asian monsoon: Links to solar changes and North Atlantic climate. Science 308, 854–857 (2005). 15879216 10.1126/science.1106296

[b18] Cheng H. *et al.* Ice Age Terminations. Science 326, 248–252 (2009). 19815769 10.1126/science.1177840

[b19] LeGrande A. & Schmidt G. Sources of Holocene variability of oxygen isotopes in paleoclimate archives. Clim. Past 5, 441–455 (2009).

[b20] Pausata F. S. R., Battisti D. S., Nisancioglu K. H. & Bitz C. M. Chinese stalagmite delta O-18 controlled by changes in the Indian monsoon during a simulated Heinrich event. Nat. Geosci. 4, 474–480 (2011).

[b21] Yang X. *et al.* Holocene stalagmite δ^18^O records in the East Asian monsoon region and their correlation with those in the Indian monsoon region. Holocene 24, 1657–1664 (2014).

[b22] Guo Z. T. *et al.* Summer monsoon variations over the last 1.2 Ma from the weathering of loess-soil sequences in China. Geophys. Res. Lett. 27, 1751–1754 (2000).

[b23] Maher B. A. & Hu M. Y. A high-resolution record of Holocene rainfall variations from the western Chinese Loess Plateau: antiphase behaviour of the African/Indian and East Asian summer monsoons. Holocene 16, 309–319 (2006).

[b24] Lu H. Y. *et al.* Variation of East Asian monsoon precipitation during the past 21 ka and potential CO_2_ forcing. Geology 41, 1023–1026 (2013).

[b25] Zhou T., Gong D., Li J. & Li B. Detecting and understanding the multi-decadal variability of the East Asian summer monsoon recent progress and state of affairs. Meteorol. Z. 18, 455–467 (2009).

[b26] Liu Z. Y. *et al.* Chinese cave records and the East Asia Summer Monsoon. Quat. Sci. Rev. 83, 115–128 (2014).

[b27] Berger A. & Loutre M. F. Insolation values for the climate of the last 10 million years. Quat. Sci. Rev. 10, 297–317 (1991).

[b28] McManus J. F., Francois R., Gherardi J. M., Keigwin L. D. & Brown-Leger S. Collapse and rapid resumption of Atlantic meridional circulation linked to deglacial climate changes. Nature 428, 834–837 (2004). 15103371 10.1038/nature02494

[b29] Praetorius S. K., McManus J. F., Oppo D. W. & Curry W. B. Episodic reductions in bottom-water currents since the last ice age. Nat. Geosci. 1, 449–452 (2008).

[b30] Moy C. M., Seltzer G. O., Rodbell D. T. & Anderson D. M. Variability of El Niño/Southern Oscillation activity at millennial timescales during the Holocene epoch. Nature 420, 162–165 (2002). 12432388 10.1038/nature01194

[b31] Zhang H., Wang S. & Cao Z. Discussion on the historical stage division of basin ecological safety - Taking Fenhe River upstream as the subjects. J. Taiyuan Norm. Univ. 6, 1–5 (2007) (In Chinese).

[b32] Telford R. J. & Birks H. J. B. A novel method for assessing the statistical significance of quantitative reconstructions inferred from biotic assemblages. Quat. Sci. Rev. 30, 1272–1278 (2011).

[b33] Reimer P. J. *et al.* IntCal09 and Marine09 radiocarbon age calibration curves, 0-50,000 years cal BP. Radiocarbon 51, 1111–1150 (2009).

[b34] Chen F. H. *et al.* Environmental magnetic studies of sediment cores from Gonghai Lake: implications for monsoon evolution in North China during the late glacial and Holocene. J Paleolimnol 49, 447–464 (2013).

[b35] Birks H. J. B., Heiri O., Seppä H. & Bjune A. E. Strengths and weaknesses of quantitative climate reconstructions based on late-Quaternary biological proxies. Open Ecol. J. 3, 68–110 (2010).

[b36] Shakun J. D. *et al.* Global warming preceded by increasing carbon dioxide concentrations during the last deglaciation. Nature 484, 49–54 (2012). 22481357 10.1038/nature10915

[b37] Fleitmann D. *et al.* Evidence for a widespread climatic anomaly at around 9.2 ka before present. Paleoceanography 23, PA1102, doi:10.1029/2007PA001519 (2008).

[b38] Yang B. *et al.* A 3,500-year tree-ring record of annual precipitation on the northeastern Tibetan Plateau. Proc. Natl. Acad. Sci. 111, 2903–2908 (2014). 24516152 10.1073/pnas.1319238111 PMC3939907

[b39] Mann M. E. *et al.* Global signatures and dynamical origins of the Little Ice Age and Medieval Climate Anomaly. Science 326, 1256–1260 (2009). 19965474 10.1126/science.1177303

[b40] Wang H., Chen J., Zhang X. & Chen F. Palaeosol development in the Chinese Loess Plateau as an indicator of the strength of the East Asian summer monsoon: Evidence for a mid-Holocene maximum. Quat. Int. 334-335, 155–164 (2014).

[b41] Shen J., Liu. X. Q., Wang S. M. & Ryo M. Palaeoclimatic changes in the Qinghai Lake area during the last 18,000 years. Quat. Int. 136, 131–140 (2005).

[b42] Zhao Y., Chen F. H., Zhou A. F., Yu Z. C. & Zhang K. Vegetation history, climate change and human activities over the last 6200 years on the Liupan Mountains in the southwestern Loess Plateau in central China. Palaeogeogr Palaeoclimatol Palaeoecol 293, 197–205 (2010).

[b43] Barber D. C. *et al.* Forcing of the cold event of 8,200 years ago by catastrophic drainage of Laurentide lakes. Nature 400, 344–348 (1999).

[b44] Carlson A. E. *et al.* Rapid early Holocene deglaciation of the Laurentide ice sheet. Nat. Geosci. 1, 620–624 (2008).

[b45] Yu S. Y. *et al.* Freshwater Outburst from Lake Superior as a Trigger for the Cold Event 9300 Years Ago. Science 328, 1262–1266 (2010). 20430972 10.1126/science.1187860

[b46] Liu Y. H. *et al.* Links between the East Asian monsoon and North Atlantic climate during the 8,200 year event. Nat. Geosci. 6, 117–120 (2013).

[b47] Wang L., Sarnthein M., Grootes P. M. & Erlenkeuser H. Millennial reoccurrence of century-scale abrupt events of East Asian Monsoon: A possible heat conveyor for the global deglaciation. Paleoceanography 14, 725–731 (1999).

[b48] Zhang R. & Delworth T. L. Simulated Tropical Response to a Substantial Weakening of the Atlantic Thermohaline Circulation. J. Clim. 18, 1853–1860 (2005).

[b49] Lisiecki L. E. & Raymo M. E. A Pliocene-Pleistocene stack of 57 globally distributed benthic δ^18^O records. Paleoceanography 20, PA1003, doi:10.1029/2004PA00107 (2005).

[b50] Stanford J. *et al.* Sea-level probability for the last deglaciation: A statistical analysis of far-field records. Glob. Planet. Chang. 79, 193–203 (2011).

[b51] Chiang J. C. H. & Sobel A. H.. Tropical tropospheric temperature variations caused by ENSO and their Influence on the remote tropical climate. J. Clim. , 15, 2616–2631 (2002).

[b52] Xie S. P. *et al.* Indian Ocean capacitor effect on Indo–Western Pacific climate during the summer following El Niño. J. Clim. , 22, 730–747 (2009).

[b53] Zhou T. J. & Zou L. Understanding the predictability of East Asian summer monsoon from the reproduction of land-sea thermal contrast change in AMIP-type simulation. J. Clim. , 23, 6009–6026 (2010).

[b54] Dykoski C. A. *et al.* A high-resolution, absolute-dated Holocene and deglacial Asian monsoon record from Dongge Cave, China. Earth Planet. Sci. Lett. 233, 71–86 (2005).

[b55] Marcott S. A., Shakun J. D., Clark P. U. & Mix A. C. A reconstruction of regional and global temperature for the past 11,300 years. Science 339, 1198–1201 (2013). 23471405 10.1126/science.1228026

[b56] Liu J., Wang B., Cane M. A., Yim S. Y. & Lee J. Y. Divergent global precipitation changes induced by natural versus anthropogenic forcing. Nature 493, 656–659 (2013). 23364744 10.1038/nature11784

[b57] Faegri K., Kaland P. E. & Krzywinski K. Text Book of Pollen Analysis (John Wiley & Sons Inc, London, 1989).

[b58] Wang F. X., Qian N. F., Zhang Y. L. Pollen Flora of China (Science Press: Beijing, 1995).

[b59] Grimm E. C. TILIA Version 2.0.b.4 (Springfield, IL: Illinois State Museum, 1993).

[b60] Grimm E. C. CONISS: a FORTRAN 77 program for stratigraphically constrained cluster analysis by the method of incremental sum of squares. Comput Geosci 13, 13–15 (1987).

[b61] Ramsey C. B. Deposition models for chronological records. Quat. Sci. Rev. 27, 42–60 (2008).

[b62] Zheng Z. *et al.* Comparison of climatic threshold of geographical distribution between dominant plants and surface pollen in China. Sci. China Ser. D. Earth Sci. 51, 1107–1120 (2008).

[b63] Xu Q. H., Xiao J. L., Li Y. C., Tian F. & Nakagawa T. Pollen-based quantitative reconstruction of Holocene climate changes in the Daihai Lake area, Inner Mongolia, China. J. Clim. 23, 2856–2868 (2010).

[b64] Zheng Y. *et al.* Palynological and satellite-based MODIS observations of modern vegetational gradients in China. Quat. Int. 218, 190–201 (2010).

[b65] Lu H. Y. *et al.* Modern pollen distributions in Qinghai-Tibetan Plateau and the development of transfer functions for reconstructing Holocene environmental changes. Quat. Sci. Rev. 30, 947–966 (2011).

[b66] Birks H. J. B. Numerical tools in palaeolimnology - Progress, potentialities, and problems. J. Paleolimnol. 20, 307–332 (1998).

[b67] Lorenz S. J. & Lohmann G. Acceleration technique for Milankovitch type forcing in a coupled atmosphere-ocean circulation model: method and application for the Holocene. Clim. Dyn. 23, 727–743 (2004).

[b68] Jin L., Chen F., Morrill C., Otto-Bliesner B. L. & Rosenbloom N. Causes of early Holocene desertification in arid central Asia. Clim. Dyn. 38, 1577–1591 (2012).

[b69] Doty B. The Grid Analysis and Display System (GrADS) version 1.5.1.12. (Fairfax, VA: Center for Ocean-Land-Atmosphere Studies, 1995).

