# Supplementary material for: East Asian summer monsoon precipitation variability since the last deglaciation
Source: Sci Rep. 2015 Jun 18;5:11186. doi: 10.1038/srep11186 (PMC4471663; doi:10.1038/srep11186)
Supplement: Supplementary Information [file srep11186-s1.pdf]

Supplementary Information

East Asian summer monsoon precipitation variability since the last deglaciation

Fahu Chen[1,]*, Qinghai Xu[2], Jianhui Chen[1], H. John B. Birks[3,4], Jianbao Liu[1], Shengrui Zhang[2], Liya Jin[1], Chengbang An[1], Richard J. Telford[3], Xianyong Cao[2], Zongli Wang[1], Xiaojian Zhang[1], Kandasamy Selvaraj[1], Houyuan Lu[5], Yuecong Li[2], Zhuo Zheng[6], Haipeng Wang[1], Aifeng Zhou[1], Guanghui Dong[1], Jiawu Zhang[1], Xiaozhong Huang[1], Jan Bloemendal[7], Zhiguo Rao[1]

Supplementary Text

Text S1: Pollen source area

It has been suggested that the pollen source area for lakes in forest region with a radius smaller than 750 m (radius of the Gonghai Lake is smaller than 400 m) is generally not exceeding 3 km[S1,S2]. The modern vegetation around Lake Gonghai is grass and shrub dominated by *Artemisia*, Chenopodiaceae and *Hippophae*, while the forest vegetation on Guancen Mountain (potential regional pollen source area, 5-30 km from Gonghai Lake) is mainly *Picea* and *Larix*. The pollen assemblages of the top layers of GH09B are mainly composed of herb pollen such as *Artemisia* and Chenopodiaceae with extremely low (mostly lower than 10%) content of tree pollens, further indicating that the fossil pollens in Gonghai Lake sediments represent vegetation compositions around the lake basin[S3]. Given both the dominance of regional zonal vegetation (very few intra-zonal taxa) and the significant altitudinal zonation around the Gonghai Lake, the stratigraphic pollen data can thus be used to reflect the regional vegetation succession and climatic changes.

Text S2: Pollen diagram description

The pollen assemblages are dominated by arboreal pollen types including *Pinus*, *Picea*, *Betula*, *Quercus* and *Ulmus*. The herb pollen taxa are dominated by *Artemisia*, Chenopodiaceae and Gramineae; they exhibit a higher diversity than the tree pollen types. According to CONISS, five major zones can be delimited (Fig. S6):

Pollen Zone 1 (14.7–11.1 ka, 9.42–7.46 m)
This zone is dominated by *Artemisia*, Chenopodiaceae and *Betula*, with higher percentages of dry-land taxa including *Hippophae* and *Ephedra*. Two subzones are defined, which may correspond to the Bølling-Allerød interstadial (GH-1a) and the Younger-Dryas event (GH-1b). Subzone GH-1a has higher percentages of *Pinus*, *Picea*, *Ulmus* and *Quercus* and also has more desert pollen taxa, including *Ephedra* and Chenopodiaceae, compared to subzone GH-1b. High percentages of hydrophytes in this zone also indicate a shift from a terrestrial to a shallow aquatic environment, with fluvial sediment inputs below 9.42 m and lacustrine sediment above. Subzone GH-1b is characterised by rapidly increasing *Artemisia* and *Betula* pollen and decreasing herb pollen values.

Pollen Zone 2 (11.1–7.3 ka, 7.46–6.24 m)
There are two subzones in this interval. Subzone GH-2a (11.1–9.6 ka) is characterised by increasing tree pollen frequencies and declining herb pollen frequencies. *Ulmus* and *Ostryopsis* exhibit peak values for the entire zone, and in general the pollen assemblages indicate the establishment of forest near the site. In Subzone GH-2b (9.6–7.3 ka), *Pinus and Quercus* increase and there is a peak in *Picea*. *Ulmus* and

Betula values are somewhat reduced.

Pollen Zone 3 (7.3–5.0 ka, 6.24–5.59 m)
This zone is characterised by peak *Quercus* and total tree pollen frequencies. It reflects stable forest vegetation consisting mainly of conifers and mixed broadleaf trees dominated by *Quercus*.

Pollen Zone 4 (5.0–1.6 ka, 5.59–3.78 m)
This zone is divided into two subzones. In Subzone GH-4a (5.0–2.8 ka) *Pinus* dominates at the expense of *Quercus*, indicating a *Pinus*-dominated mixed conifer and broadleaved forest. Subzone GH-4b (2.8–1.6 ka), is characterised by decreasing tree pollen and increasing herb pollen frequencies, probably indicating a drying of the climate.

Pollen Zone 5 (1.6 ka–Present, 3.78–0.00 m)
This zone exhibits low tree pollen and the highest herb pollen frequencies, suggesting a significant change from a forest to a forest-grassland environment. There was some forest recovery during the Medieval Warm Period at around 600–800 cal yr BP; however, very low tree pollen frequencies occurred during the LIA. The presence of cereal pollen types (less than 4%) indicates some human impact on the vegetation during this period; however, documentary evidence suggests that intensive deforestation in this region did not occur until ~600 cal yr BP (Ming Dynasty)[S4].

Text S3: Cultural evolution
Neolithic cultural evolution in the Yellow River valley[S5,S6] corresponds closely to the precipitation variations reconstructed from the Gonghai Lake pollen record (Fig. S10). Pre-Yangshao cultures were widely scattered with a small number sites during ca. 8.3–7 ka when precipitation was lower than during the subsequent two millennia. The Yangshao culture expanded rapidly both in sites numbers and occupied areas, and became the prevailing cultural system from 7–5 ka when precipitation was highest and stable. During the Post-Yangshao period (5–4 ka), when precipitation began to decrease, it disintegrated into many different cultures.

Supplementary Figures

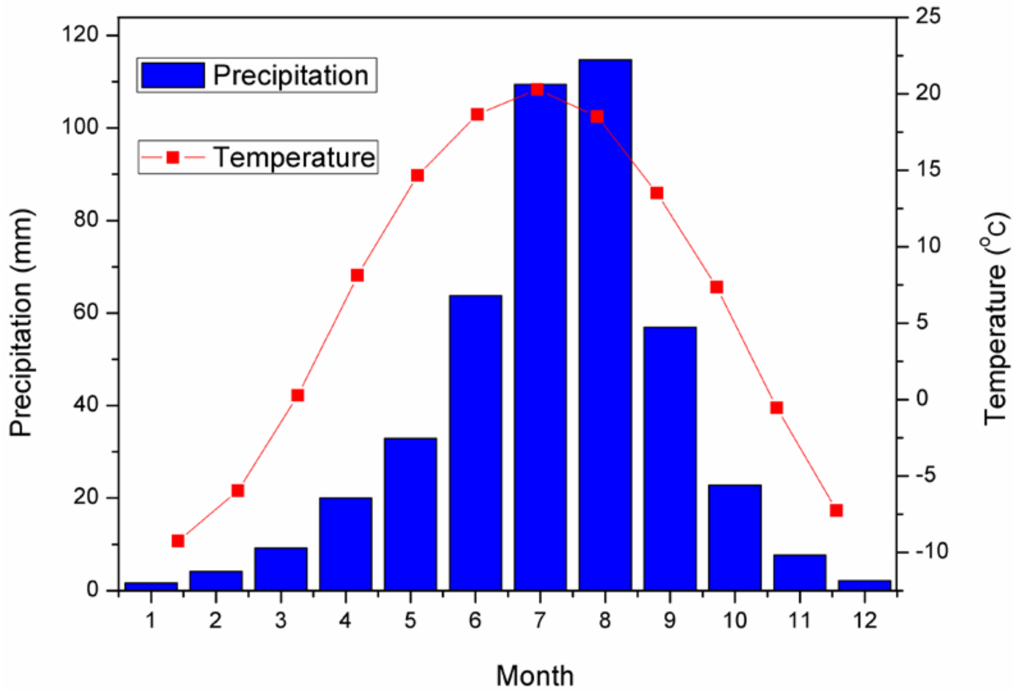

Figure S1. Instrumental climate data (AD 1959–2011) from Ningwu Station, Shanxi, China (data from Chinese Meteorological Administration).

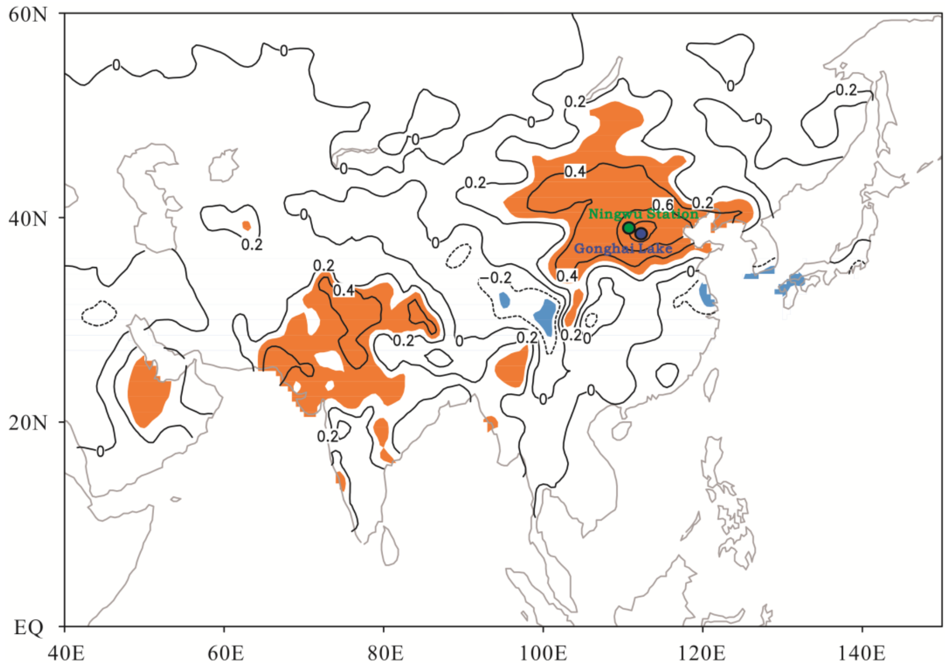

Figure S2. Correlation coefficients between precipitation at Ningwu Station (solid green dot) and NOAA/NCEP Climate Prediction Centre's precipitation reconstruction over land (PRECL), data from AD 1959 to 2011. Shaded area indicates correlations which are significant at the 95% confidence level. The position of Gonghai Lake is marked by the solid blue dot, located in a zone characterised by a sensitive response of precipitation to EASM variability[S7].

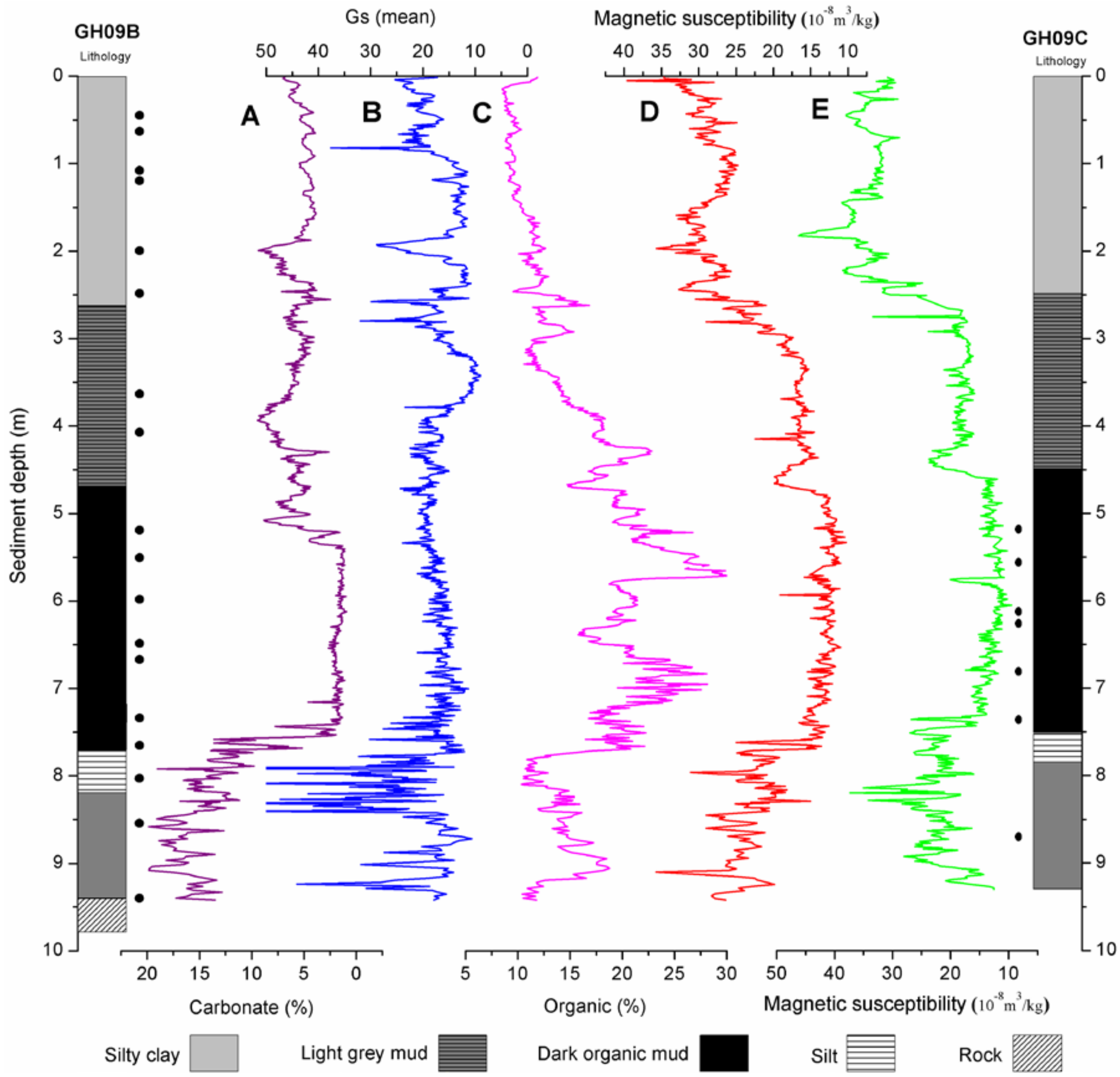

Figure S3. Simplified stratigraphy and correlation of individual sediment cores from Gonghai Lake. Curves (*A*)–(*D*) are from core GH09B and curve (*E*) from GH09C. The magnetic susceptibility records of cores GH09B (*D*) and GH09C (*E*) are easily correlated.

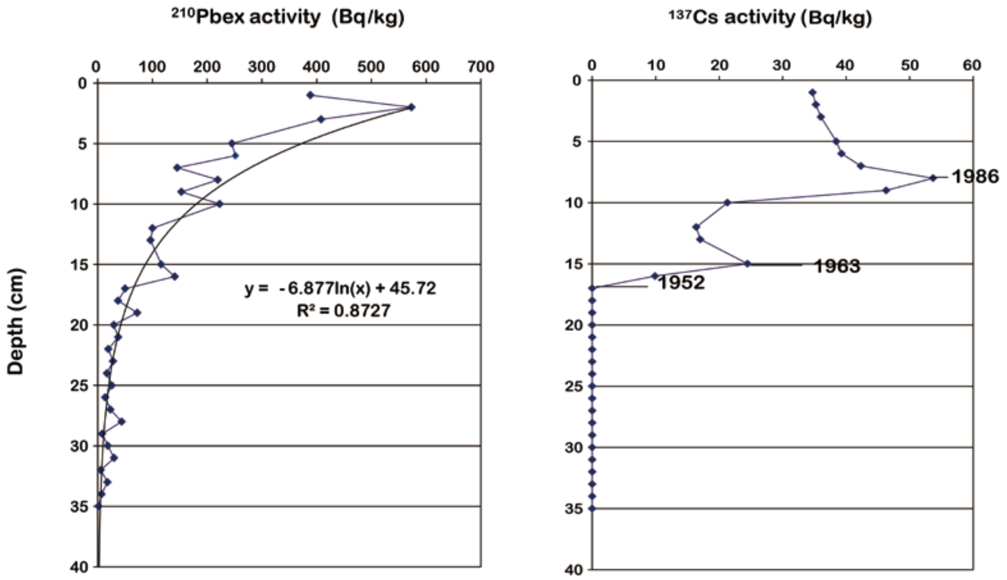

Figure S4. ^{210}Pb/^{137}Cs dating model for core GH09B. The Constant Rate of Supply (CRS) model was used[S8]. The excess ^{210}Pb activity exhibits a simple exponential relationship with depth. From the ^{210}Pb profile, the age of the ^{137}Cs peaks at 17 cm, 15 cm, and 8 cm are estimated as AD 1952, AD 1963, and AD 1986, respectively. All of these are very close to the presumed corresponding atmospheric maxima in ^{137}Cs, validating the accuracy of the ^{210}Pb dating. For this reason we use ^{210}Pb as the primary age-control for the upper 35 cm of the core.

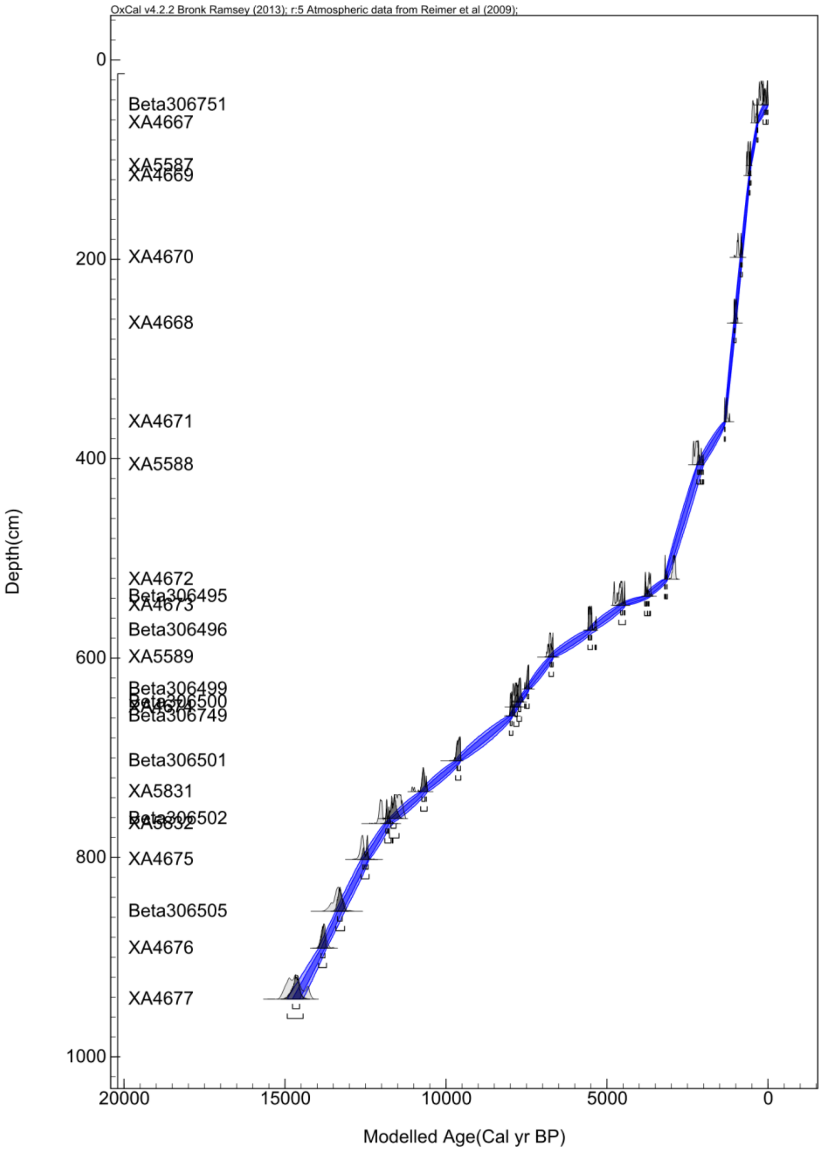

Figure S5. Age-depth model for Gonghai Lake core GH09B.

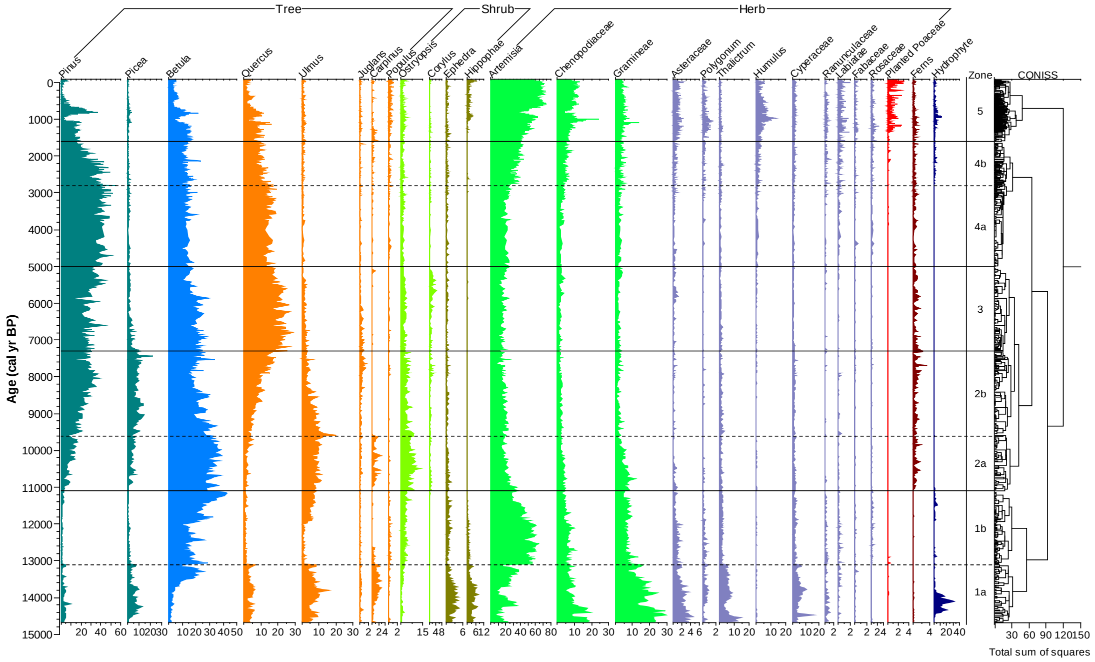

Figure S6. Pollen diagram from Gonghai Lake.

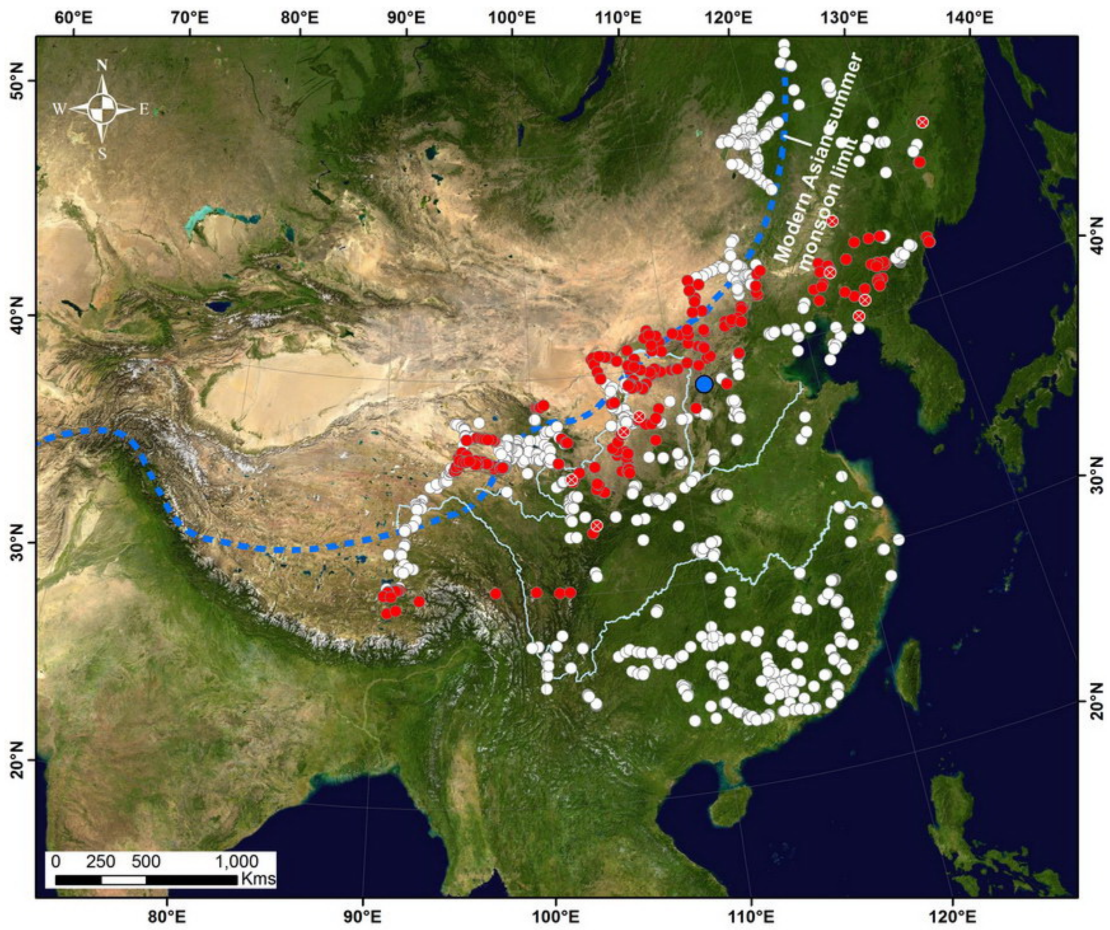

Figure S7. The spatial distribution of modern surface samples used in this study. The white dots are the 1860 samples remaining after removing those from tropical and arid regions, and those which could potentially be significantly influenced by human activity (i.e. close to roads, farmlands, etc.) according to the original field notes. The red dots are the 509 samples used for development of the calibration function; outliers are indicated by crosses. The blue dot marks the location of Gonghai Lake. It is evident from the satellite image that the red dots are located at the transitional zone between the vegetated region and relatively barren land. The map was generated using ESRI ArcGIS v9.3[S9].

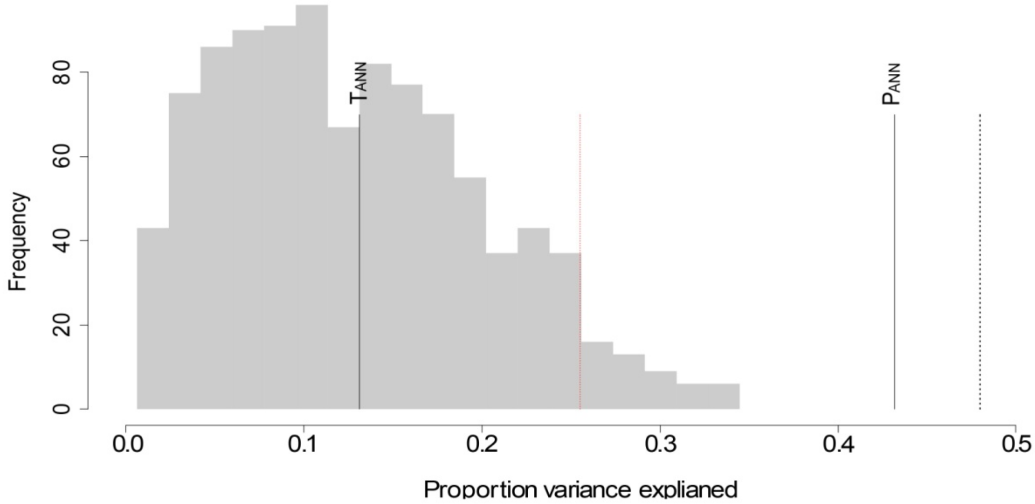

Figure S8. Histogram of the proportion of variance in the GH09B pollen record explained by 999 calibration functions (WAPLS-2) trained with random environmental data. Solid black lines indicate the proportion of variance explained by our T_{ANN} and P_{ANN} transfer functions, from right to left, respectively. The red dotted line indicates the proportion of variance below which 95% of the random data-trained calibration functions could be explained. The black dotted line indicates the proportion of variance explained by the first axis of a PCA of the fossil data. It is showed that the P_{ANN} reconstruction based on the WAPLS-2 model and stratigraphical pollen data from core GH09B explains more of the variation in the fossil data than 99% of reconstructions (p = 0.001) derived from calibration functions trained on random environmental data, whereas the T_{ANN} reconstruction is not statistically significant (p = 0.434).

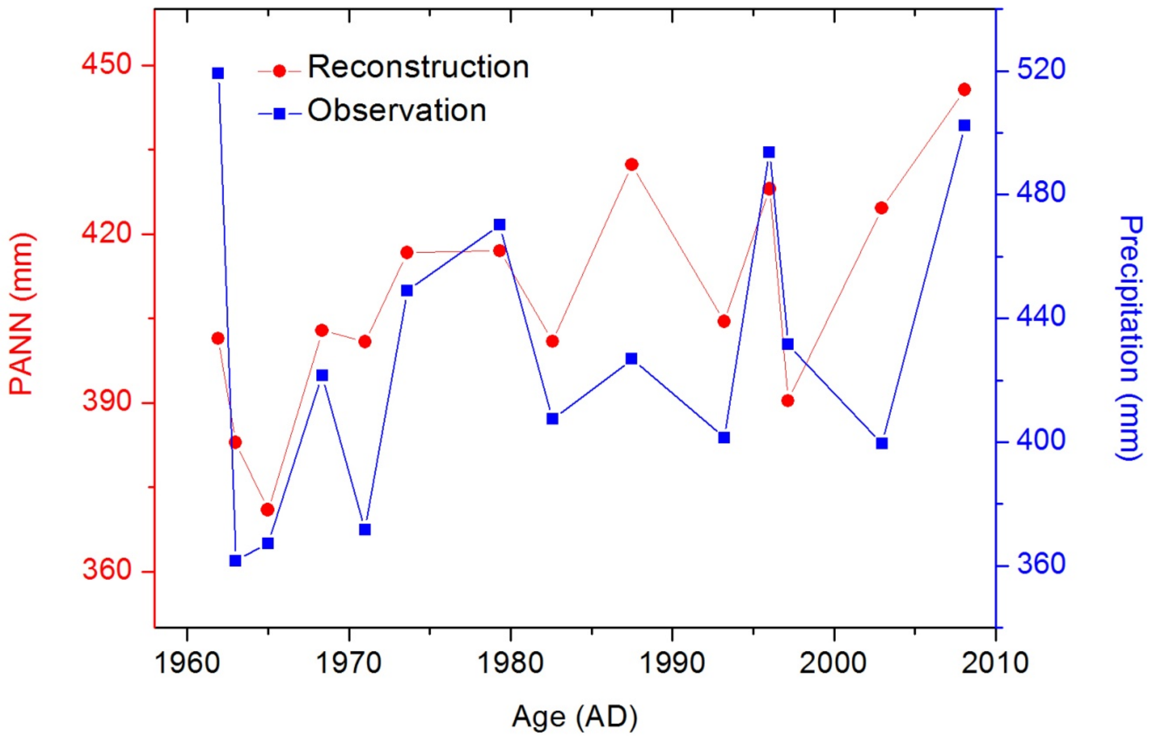

Figure S9. Comparison of P_{ANN} reconstruction and observations from AD 1962 to 2008. The observational P_{ANN} values were averaged according to the chronologically unevenly-distributed fourteen reconstructed P_{ANN} values.

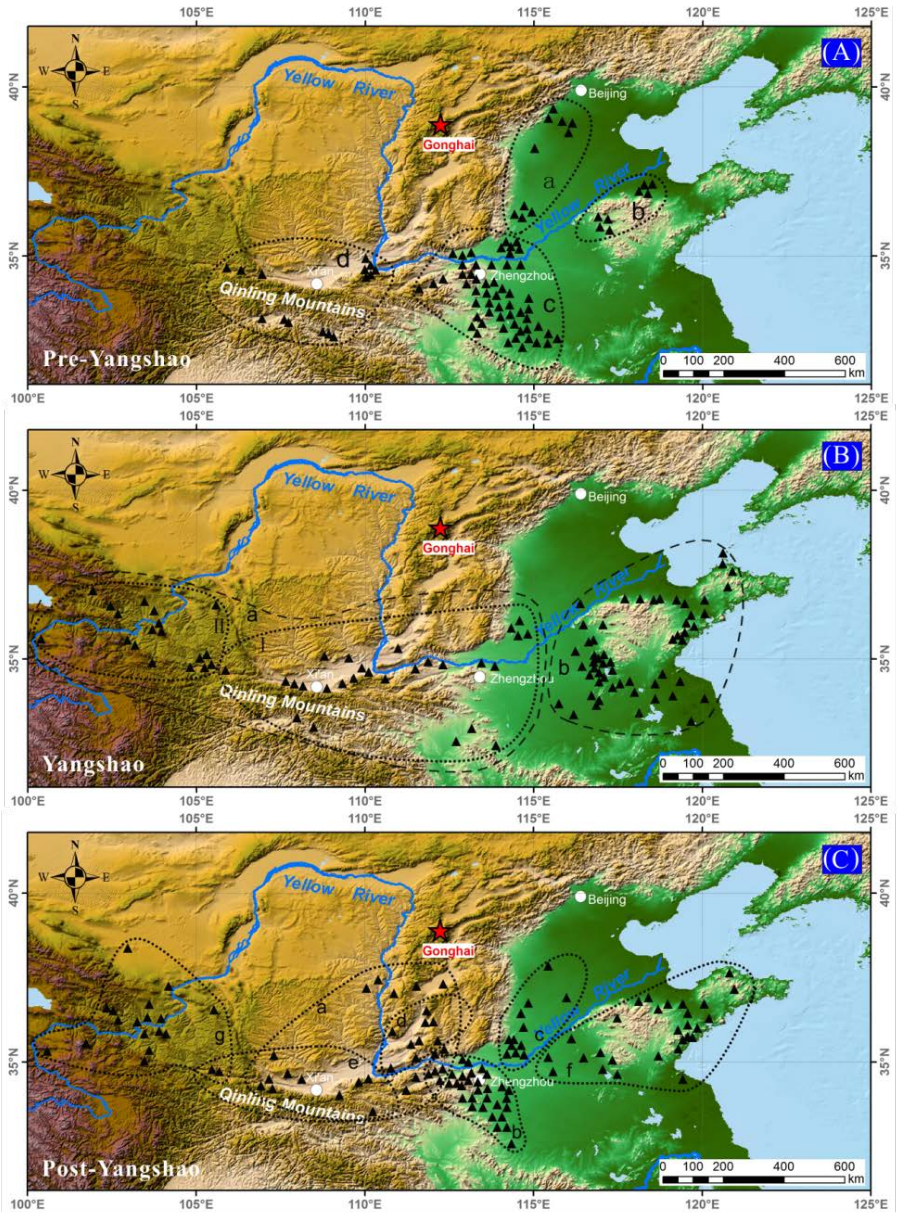

Figure S10. Spatial and temporal pattern of cultural evolution during the Neolithic period in the Yellow River region, North China[S5,S6]. The occupied areas of different Neolithic cultures (dotted lines) are defined by the distributions of their excavated sites (black triangles). (*A*) Pre-Yangshao cultural period. a. Cishan culture (8.1–7.8 ka); b. Houli culture (8.3–7.4 ka); c. Peiligang culture (8.2–7.5 ka); d. Dadiwan-Laoguantai culture (7.9–7.0 ka). (*B*) Yangshao cultural period. a. Yangshao cultural system (7.0–5.0 ka); I. Yangshao culture (7.0–4.9 ka); II. Shilingxia-Majiayao culture (6.0–4.9 ka); b. Beixin-Dawenkou culture (7.4–4.6 ka). (*C*) Post-Yangshao culture period. a. The second period of Miaodigou culture (5.0–4.6 ka); b. The third period of Wangwan culture (4.6–3.9 ka); c. The second period of Hougang culture (4.6–4.0 ka); d. Taosi culture (4.6–4.0 ka); e. Keshengzhuang culture (4.6–4.0 ka); f. Longshan culture (4.6–4.0 ka); g. Banshan-Machang culture (4.5–4.0 ka). The base map was generated by ESRI ArcGIS v9.3[S9] using the ETOPO1 global relief model[S10].

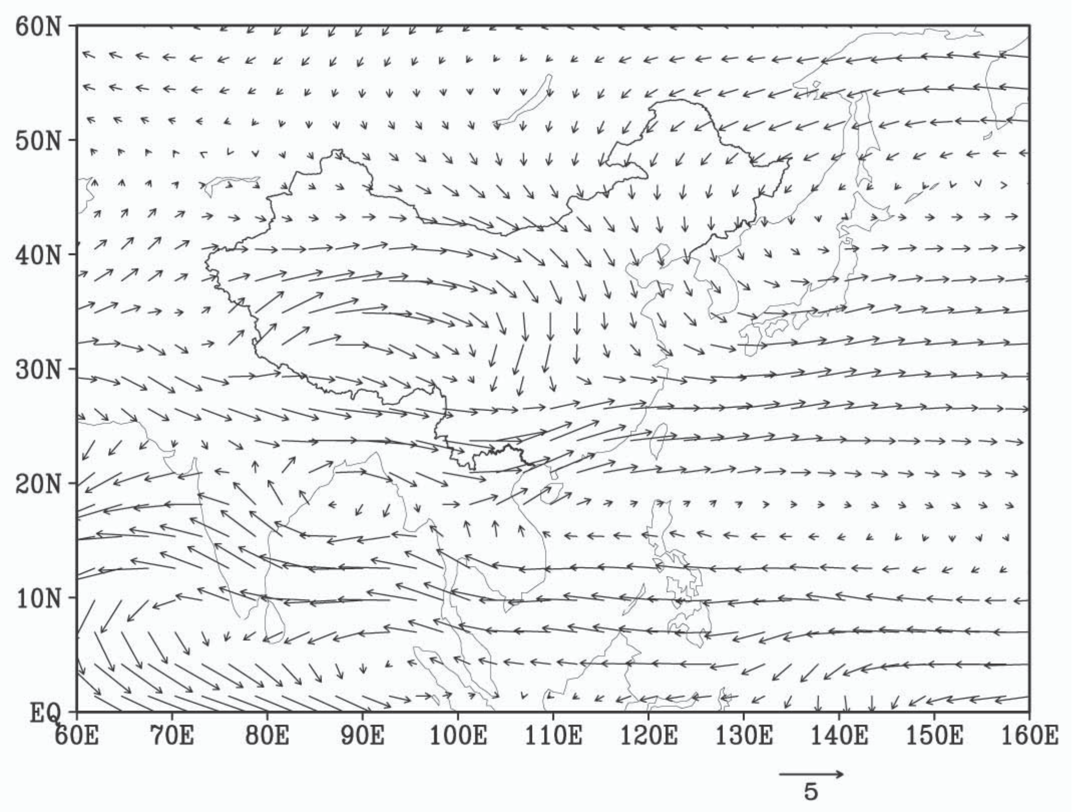

Figure S11. The difference of 850hPa wind fields (in m/s) between Exp_8.5 ka MELTICE and Exp_8.5 ka, indicating the effect of the Laurentide Ice Sheet and associated melt-water input.

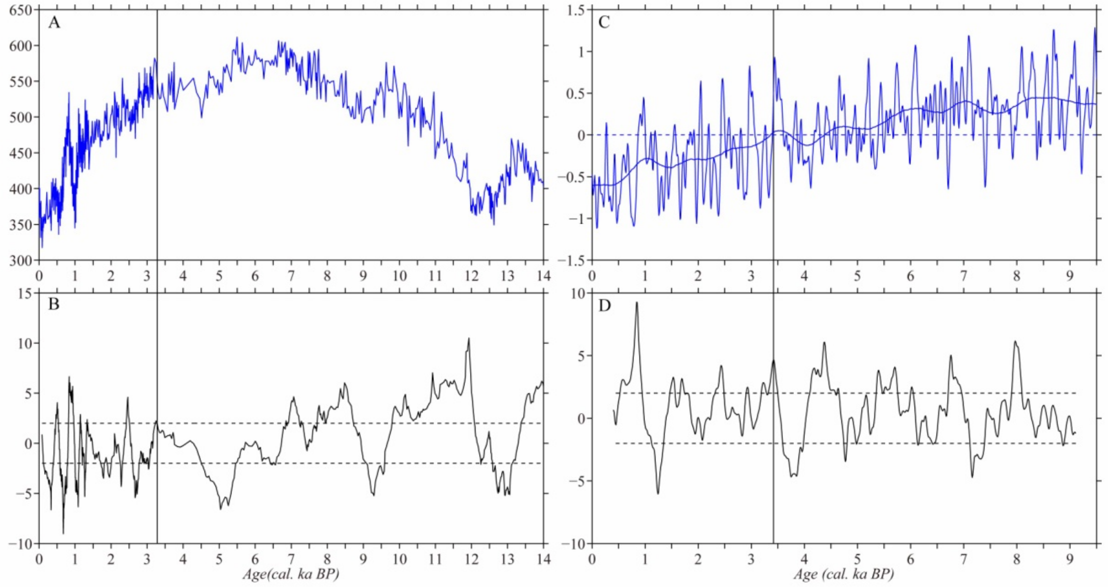

Figure S12. (*A*) The proxy-based EASM precipitation. (*B*) The moving *T*-test of the proxy-based EASM precipitation with subseries lengths L=18. (*C*) Simulated EASM index during the Holocene, defined as the evolution of summer (JJA) precipitation around Gonghai Lake averaged over the region (35º–45ºN, 105º–120ºE) from the Kiel Climate Model transient climate simulation for the last 9.5 ka with a 9-point and 99-point running mean, only considering orbital forcings. (*D*) The moving *T*-test of the 9-point filtered EASM index (modelled) with subseries lengths L=40. Horizontal black dash lines denote the 95% confidence level; vertical lines in (*B*) and (*D*) indicate possible abrupt points. Both the proxy-based and model-based EASM index indicate an abrupt monsoon retreat at 3.3 ka.

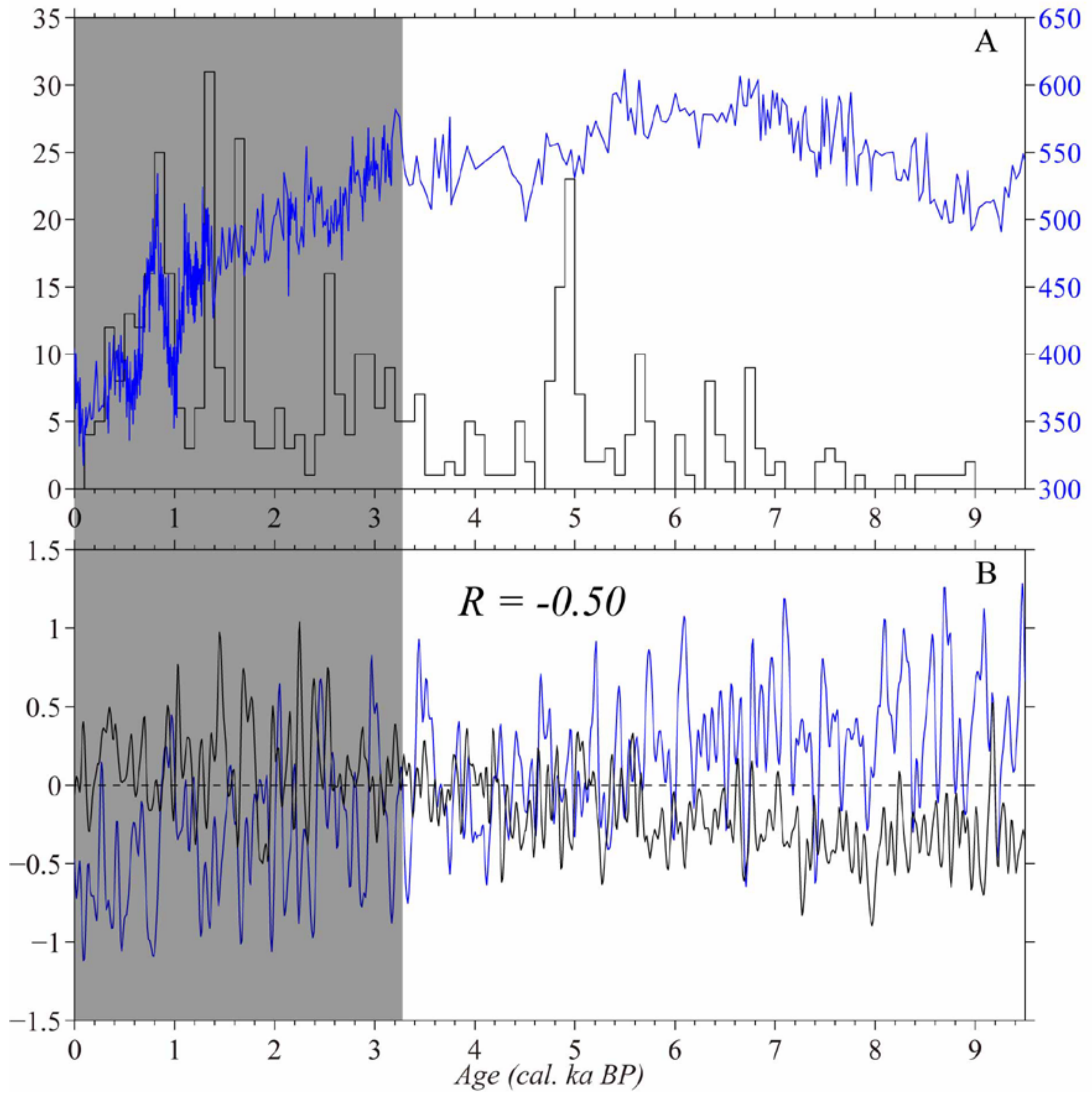

Figure S13. Comparison of (*A*) proxy and (*B*) model based EASM index (blue line) and ENSO[S11]. The shaded area shows a relatively active ENSO during the late Holocene.

Supplementary Tables

Table S1. Radiocarbon dates of terrestrial plant-macrofossil samples for core GH09B and GH09C from Gonghai Lake, using for chronology construction.

**Lab ID**	**Sample No.**	**Depth (cm)**	**Material**	$\delta^{13}C$ ‰	**Conventional Age (1σ, BP yr)**	**Calibrated Age(1σ, Cal yr BP)**
Beta306751	GHB1-35	0.46	Stem	-25.9	150±30	0-282
XA4667	GHB1-48	0.63	Leaf	-24.9	368±23	332-498
XA5587	GHB1-82	1.07	Leaf	-15.7	570±25	540-628
XA4669	GHB1-89	1.16	Stem	-30.7	665±34	565-668
XA4670	GHB1-153	1.99	Stem	-30.9	1005±40	804-963
XA4668	GHB1-191	2.49	Stem	-30.4	1102±30	968-1053
XA4671	GHB2-76	3.63	Stem	-25.4	1329±25	1194-1294
XA5588	GHB2-112	4.06	Leaf	-28.7	2231±35	2159-2325
XA4672	GHB2-220	5.21	Stem	-24.3	2821±29	2878-2956
Beta306495	GHC2-257	5.38	Stem	-21.6	3440±30	3640-3813
XA4673	GHB2-245	5.48	Stem	-34.1	4089±39	4455-4797
Beta306496	GHC3-21	5.72	Stem	-22.2	4760±40	5470-5583
XA5589	GHB3-34	6.00	Leaf	-21.3	5944±36	6727-6844
Beta306499	GHC3-70	6.31	Stem	-25.7	6550±40	7427-7485
Beta306500	GHC3-81	6.44	Stem	-22.0	6890±50	7674-7785
XA4674	GHB3-82	6.49	Leaf	-28.2	7001±54	7765-7930
Beta306749	GHB3-90	6.58	Stem	-17.9	7120±40	7876-7995
Beta306501	GHC3-128	7.03	Stem	-27.0	8670±40	9550-9659
XA5831	GHB3-161	7.35	Leaf	-24.2	9464±42	10602-10758
Beta306502	GHC3-174	7.61	Stem	-14.0	10020±50	11360-11690
XA5832	GHB3-189	7.66	Leaf	-25.9	10252±39	11839-12084
XA4675	GHB3-222	8.02	Stem	-16.5	10667±83	12655-12820
Beta306505	GHC4-43	8.54	Stem	-20.0	11930±50	13725-13859
XA4676	GHB4-6	8.91	Stem	-33.3	11490±140	13203-13477
XA4677	GHB4-45	9.42	Leaf	-29.2	12518±49	14487-14883

Table S2. Bootstrap-derived performance statistics for the weighted-averaging partial least squares (WAPLS) calibration function (N_{boot} = 1,000). RMSEP stands for root mean squared error of prediction.

**WAPLS Component**	**RMSEP (mm)**	r^2	**Average Bias (mm)**	**Maximum Bias (mm)**	**ΔRMSEP (%)**	***P***
1	95.88	0.79	1.56	189.18	NA	NA
2	85.85	0.84	3.45	147.44	-10.46	0.000
3	86.86	0.84	0.27	109.36	1.17	0.240
4	87.72	0.85	1.70	106.89	0.99	0.357
5	90.03	0.84	1.57	93.03	2.63	0.697

Table S3. Transient simulation boundary conditions.

	Eccentricity	Obliquity	Precession	CO_2(ppm)	CH_4(ppb)	N_2O(ppb)
**H0K**	0.0167	23.4º	102º	286.2	805.6	276.7
**H9K**	0.0194	24.2º	303º	Same as H0K		
**HT**	Varying from H9K to H0K			Same as H0K		

Table S4. Sensitivity simulation boundary conditions.

	**PI**	**Exp_6 ka**	**Exp_8.5 ka**	**Exp_8.5 ka$_{ICE}$**	**Exp_8.5 ka$_{MELTICE}$**
Eccentricity	0.016724	0.018682	0.019199	Same as Exp_8.5 ka	Same as Exp_8.5 ka
Obliquity	23.45º	24.10º	24.22º	Same as Exp_8.5 ka	Same as Exp_8.5 ka
Precession	102.04º	0.87º	319.50º	Same as Exp_8.5 ka	Same as Exp_8.5 ka
CO_2 (ppm)	280	280	260	Same as Exp_8.5 ka	Same as Exp_8.5 ka
CH_4 (ppb)	760	650	660	Same as Exp_8.5 ka	Same as Exp_8.5 ka
N_2O (ppb)	270	270	260	Same as Exp_8.5 ka	Same as Exp_8.5 ka
Laurentide Ice Sheet	None	None	None	ref. S12	ref. S12
St. Lawrence meltwater flux	None	None	None	None	0.05 verdrups

Supplementary References

S1. Nielsen, A. B. & Sugita, S. Estimating relevant source area of pollen for small Danish lakes around 641 AD 1800. *Holocene* **15,** 1006-1020 (2005).

S2. Sugita, S. Pollen representation of vegetation in Quaternary sediments: theory and method in patchy vegetation. *J. of Ecol.* **82,** 881-897 (1994).

S3. Xu, Q. H. et al. Vegetation succession and Eastern Asian summer monsoon changes since the last deglaciation inferred from high resolution pollen record in Lake Gonghai, Shanxi Province, China. Submitted to *Quat. Sci. Rev.* (2015).

S4. Zhang, H., Wang, S. & Cao, Z. Discussion on the historical stage division of basin ecological safety - Taking Fenhe River upstream as the subjects. *J. Taiyuan Norm. Univ*. **6,** 1-5 (2007) (In Chinese).

S5. IA CASS (Institute of Archaeology Chinese Academy of Social Sciences). *Chinese Archaeology, Neolithic Volume*. (2010) (In Chinese).

S6. Yan, W. M. in *Research of Yangshao Culture* (ed W. M. Yan) (Cultral Relics Press, Beijing, 1989) (In Chinese).

S7. Liu, J. *et al.* Forced response of the East Asian summer rainfall over the past millennium: results from a coupled model simulation. *Clim. Dyn.* **36,** 323-336 (2011).

S8. Appleby. P. *et al.* ^{210}Pb dating by low background gamma counting. *Hydrobiologia* **141,** 21-27 (1986).

S9. ESRI (Environmental Systems Resource Institute). *ArcGIS Version 9.3* (Redlands, CA: ESRI, 2009)

S10. Amante, C. & Eakins, B. W. ETOPO1 1 Arc-Minute Global Relief Model: Procedures, Data Sources and Analysis. *NOAA Technical Memorandum NESDIS NGDC* **24**, 1-19 (2009).

S11. Moy, C. M. Seltzer, G. O., Rodbell, D. T. & Anderson, D. M. Variability of El Niño/Southern Oscillation activity at millennial timescales during the Holocene epoch. *Nature* **420**, 162-165 (2002).

S12. Peltier, W. Global glacial isostasy and the surface of the ice-age Earth: The ICE-5G (VM2) model and GRACE. *Annu. Rev. Earth Planet. Sci*. **32,** 111-149 (2004).
